# Feedback inhibition of L1 and alu retrotransposition through altered double strand break repair kinetics

**DOI:** 10.1186/1759-8753-1-22

**Published:** 2010-10-27

**Authors:** Nicholas A Wallace, Victoria P Belancio, Zach Faber, Prescott Deininger

**Affiliations:** 1 BioMedical Sciences Graduate Program, Tulane University School of Medicine, New Orleans, LA 70112, USA; 2 Department of Structural and Cellular Biology, Tulane University School of Medicine, New Orleans, LA 70112, USA; 3 Tulane Cancer Center and the Department of Epidemiology, Tulane University, New Orleans, LA 70112, USA; 4 Human Biology, Fred Hutchinson Cancer Research Center, Seattle, WA 98109, USA

## Abstract

**Background:**

Cells adapt to various chronic toxic exposures in a multitude of ways to minimize further damage and maximize their growth potential. Expression of L1 elements in the human genome can be greatly deleterious to cells, generating numerous double strand breaks (DSBs). Cells have been reported to respond to chronic DSBs by altering the repair of these breaks, including increasing the rate of homology independent DSB repair. Retrotransposition is strongly affected by proteins involved in DSB repair. Therefore, L1 expression has the potential to be a source of chronic DSBs and thus bring about the changes in cellular environment that could ultimately restrict its own retrotransposition.

**Results:**

We demonstrate that constitutive L1 expression leads to quicker DSB repair and decreases in the retrotransposition potential of L1 and other retrotransposons dependent on L1 expression for their mobility. This cellular adaptation results in reduced sensitivity to L1 induced toxicity. These effects can be induced by constitutive expression of the functional L1 ORF2 alone, but not by the constitutive expression of an L1 open reading frame 2 with mutations to its endonuclease and reverse transcriptase domains. This adaptation correlates with the relative activity of the L1 introduced into the cells.

**Conclusions:**

The increased number of DSBs resulting from constitutive expression of L1 results in a more rapid rate of repair. The cellular response to this L1 expression also results in attenuation of retrotransposition and reduced sensitivity of the cells to negative consequences of L1 ORF2 expression. The influence does not appear to be through RNA interference. We believe that the increased rate of DSB repair is the most likely cause of the attenuation of retrotransposition. These alterations act as a fail safe mechanism that allows cells to escape the toxicity associated with the unchecked L1 expression. This gives cells that overexpress L1, such as tumor cells, the ability to survive the high levels of expression. However, the increased rate of break repair may come at the cost of accuracy of repair of the lesion, resulting in increased genomic instability.

## Background

Mammalian cells often evolve adaptive responses to deal with chronic exposure to various toxic agents, including ethanol and opiates [1-4]. Cells also adapt to chronic exposure to sublethal doses of DNA double strand breaks (DSBs) through the selection of cells with altered DSB repair [1]. Typically, mammalian cells depend on a balance between two broad classes of DSB repair to ensure proper genome maintenance. Homologous repair (HR) is a process largely dependent on homology, whereas non-homologous end joining (NHEJ) is mostly independent of homology [5]. Chronic sublethal levels of DSBs cause an altered balance between these two pathways, shifting the balance towards NHEJ [1].

Long interspersed element-1 (LINE-1 or L1) is the most numerous and only currently active family of human autonomous, non-long terminal repeat (LTR) retrotransposons. They constitute 17% of the genome, with approximately 500,000 copies. Most of these L1 elements do not contain functional copies of the two open reading frames (ORF1 and ORF2) required for efficient retrotransposition [6-8]. Only around 100 L1 elements contain functional copies of both ORFs, which may allow them to contribute to DNA damage and human disease [7,9].

The L1 ORF2p has been demonstrated to contain both endonuclease and reverse transcriptase activities, which are crucial for retrotransposition [10-12]. These ORF2 domains play essential roles in target primed reverse transcription (TPRT), the proposed mechanism for the retrotransposition of L1 elements [13-15]. This mechanism predicts the transient creation of a DSB at the site of integration.

L1 expression is associated with DSB formation [16,17], and long term exposure to sublethal levels of DSBs has been associated with alterations in the repair of DNA lesions [1]. Therefore, long term exposure to L1 expression may also alter the response of a cell to DNA damage, which may influence the retrotransposition process. Retrotransposition is strongly affected by several DNA repair proteins, including excision repair cross complementing/xeroderma pigmentosum F (ERCC1/XPF), ataxia telangiectasia mutated (ATM) and p53 [16,18]. Furthermore, the DSBs caused by L1 have been implicated as a causative agent in creating chromosomal translocations normally associated with cancer [19], probably through a NHEJ mechanism.

High levels of endogenous full length L1 mRNA expression have been detected in multiple tissues and cell lines [20]. The endogenous expression of this full length L1 mRNA implies that various tissues and cell lines are exposed to chronic expression of L1 ORF2p. The expression of L1, particularly the L1 ORF2p, has been shown to result in substantially decreased cellular proliferation and increased cell death [17,21]. This toxicity is probably due to induction of DSBs associated with the expression of L1 ORF2p, because a mutation in the endonuclease domain of the L1 ORF2p greatly diminishes the L1-associated toxicity.

Using cells stably expressing both functional and non-functional L1 ORF2p, we show that cells adapt to the constitutive expression of the functional L1 ORF2p in a dose dependent manner, by repressing the retrotransposition of both L1 and Alu. Furthermore, this cellular adaptation, which limits retrotransposition, also diminishes the toxicity typically associated with the expression of L1 ORF2p. We demonstrate that cells exposed to constitutive L1 ORF2p expression have altered DNA DSB repair kinetics, which provides a potential explanation for the reduction of L1 and Alu mobilization, and the diminished toxicity associated with L1 expression.

## Results

### Constitutive L1 expression results in reduced retrotransposition

**Figure 1:**
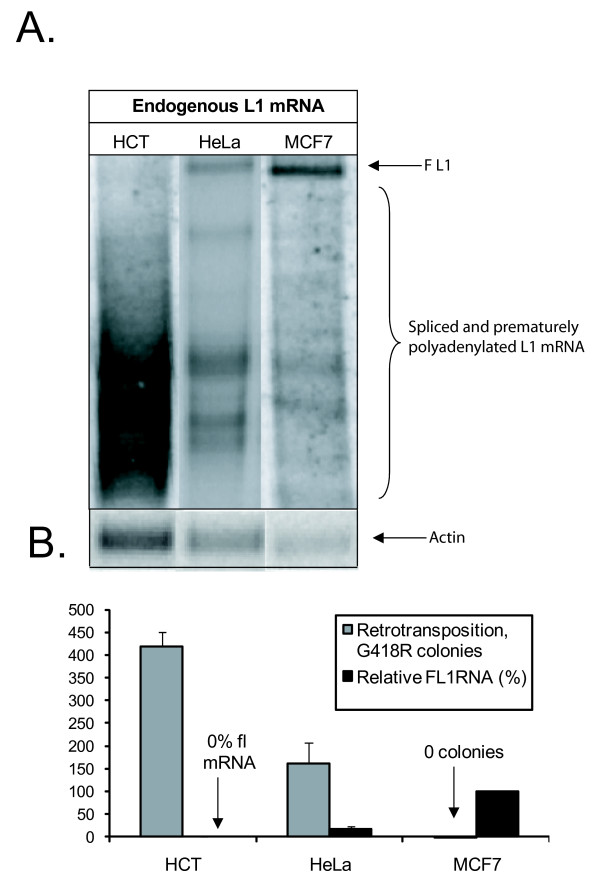
**Inverse correlation between endogenous full length L1 mRNA and retrotransposition potential**. **(A) **Northern blot of endogenous L1 mRNA (see text for method). FL1 or full length L1 mRNA, as determined by size is denoted by an arrow. L1 mRNA species previously characterized as truncated products caused by internal splicing or premature polyadenylation are indicated by a bracket [22,23]. As a loading control, the same blot was probed for β-actin RNA. **(B) **Inverse correlation between endogenous L1 mRNA Levels and retrotransposition potential. Mobilization of a tagged L1, which confers neomycin resistance to a cell upon successful retrotransposition, as described previously, [12] and the relative amount of endogenous full length L1 mRNA was measured in triplicate for HCT116, HeLa and MCF7 cells, and the means and standard deviations plotted.

Endogenous L1 mRNA is subject to splicing and premature polyadenylation [20,22,23], which results in a significant portion of the transcripts being truncated and therefore not fully functional. Northern blots of several common cancer cell lines (HCT116, HeLa and MCF7) showed that all had L1 expression but variable levels of processing, suggesting that endogenous expression of active L1 elements varies greatly between these cell lines (Figure 1A). Using a standard retrotransposition assay [12], we found an inverse correlation between the observed endogenous full length (fl)-L1 mRNA levels and L1 retrotransposition potential in these cell lines (Figure 1B).

**Figure 2:**
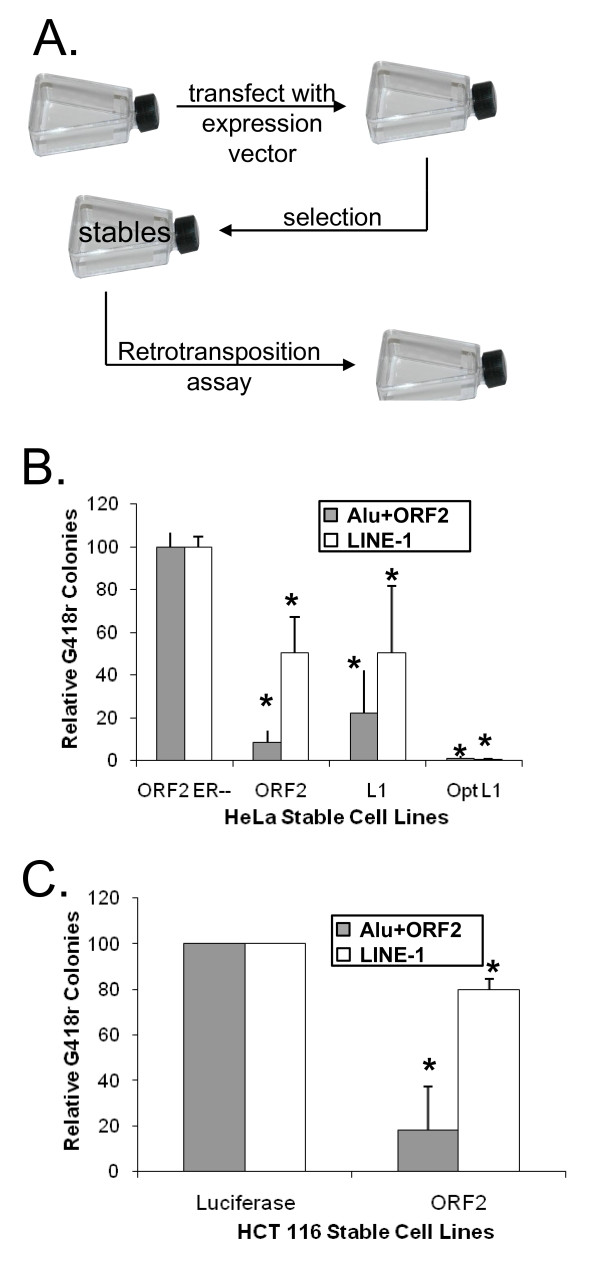
**Constitutive L1 expression results in reduced retrotransposition potential**. **(A) **Scheme for stable cell line generation. Wild-type cells were transfected with the desired expression vector. Selection was applied to assure the presence of this vector and the resulting cells were expanded under selection from colonies to a new cell line. **(B) **Constitutive L1 expression in HeLa cells results in lowered retrotransposition potential. Mobilization of a tagged L1 or a tagged Alu element, which confers neomycin resistance to a cell upon successful retrotransposition as described previously, was measured in HeLa cells selected to stably express various L1 proteins. Data are means and SD (error bars) of at least three independent measurements. Asterisks signify a significant (Student *t*-test, *P *≤ 0.05) difference from retrotransposition levels seen in HeLa open reading frame (ORF)2 ER-- cells. **(C) **Constitutive L1 Expression in HCT 116 Cells results in lowered retrotransposition potential. The mobilization of a tagged L1 or a tagged Alu element, which confers neomycin resistance to a cell upon successful retrotransposition as described previously, was measured in HCT cells selected to express stably either L1 ORF2 or luciferase. T Data are means and SD (error bars) of at least three independent measurements. Asterisks signify a significant (Student *t*-test, *P *≤ 0.005) difference from retrotransposition levels seen in HCT 116 luciferase cells.

The inverse correlation between endogenous fl-L1 mRNA levels and the retrotransposition of a tagged L1 suggested the possibility that cells can adapt to constitutive L1 expression by repressing retrotransposition. It is also possible that the varied retrotransposition rates could be caused by other cellular differences that made the cell lines differentially tolerant to the expression of L1 elements. To test the first possibility, we wished to observe the effect of L1 expression on retrotransposition in an isogenic background. We generated pools of cells that stably expressed either the entire L1 element or only the L1 ORF2 region. As negative controls, we established cells stably expressing either the L1 ORF2p with inactivating mutations in both its endonuclease and reverse transcriptase domains (L1 ORF2 ER--) or luciferase (Figure 2). Because L1 ORF2 expression generates DSBs [16], we confirmed its expression by investigating DSB levels via the formation of p53 binding protein (53BP)1 foci, a well established indicator of DSB formation [24,25]. As expected, cells stably expressing wild type L1 ORF2 showed significantly (*P *≤ 0.05) elevated levels of 53 BP1 foci compared with controls (see Additional file 1), affirming the influence of L1 ORF2 expression in these cells. We further confirmed that ORF2p was overexpressed several fold in our transfected cell lines using a western blot to ORF2p (Additional Figure 1C).

To test the effect of constitutive L1 expression on retrotransposition, we measured the retrotransposition of tagged L1 [12] and Alu [26] elements. Alu and L1 retrotransposition in both HCT116 and HeLa cells stably expressing L1 ORF2 (HeLa ORF2, HCT116 ORF2) were significantly (*P *≤ 0.05) lower than in corresponding control cells (HeLa ORF2 ER--, HCT luciferase) (Figure 2). The magnitude of repression of retrotransposition was generally greater for Alu elements than for L1 elements. Because the ORF2p level in the stable cell lines is expected to be relatively low compared with transient transfection assays and also should be variable between the various groups of cells, we cotransfected the cells with either ORF2 or full length L1 to provide relatively similar levels of ORF2 in the various cell lines to drive the retrotransposition of the tagged Alu element. The difference in the decrease in Alu retrotransposition was unaltered when Alu retrotransposition was driven by either a full length L1 or an L1 ORF2 (see Additional file 2). Decreased retrotransposition was also similar in cells stably expressing either a full length L1 element or the L1 ORF2 (Figure 2).

To determine whether increased L1 expression would result in a more pronounced phenotype, we generated HeLa cells that stably express a synthetic version of L1 that had been codon optimized to increase expression of the element (HeLa Optimized L1) (See Wallace *et al*.[21] for further description). When Alu and L1 retrotransposition in these cells was measured, both Alu and L1 retrotransposition were almost completely suppressed (Figure 2).

### Constitutive L1 expression results in reduced sensitivity to toxicity associated with transient L1 expression

**Figure 3:**
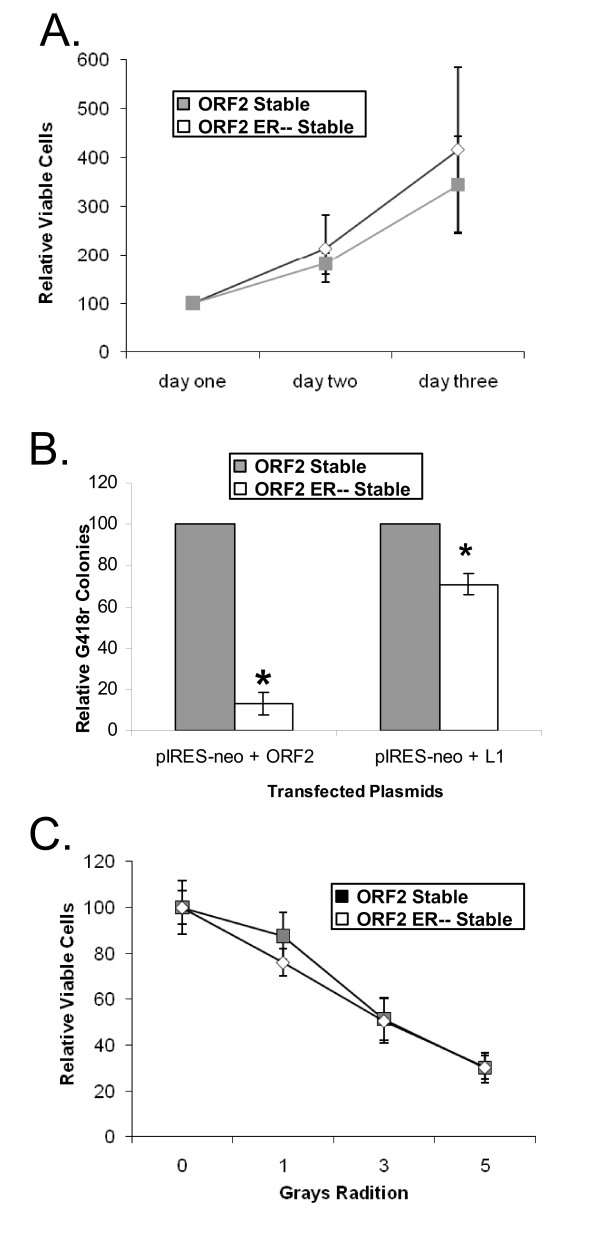
**Toxicity in cells undergoing constitutive L1 expression**. **(A) **Constitutive L1 open reading frame (ORF)2 expression in HeLa Cells results in lower sensitivity to transient L1 expression. To measure the toxicity of L1 expression, a plasmid expressing a neomycin-resistance cassette was cotransfected with a vector expressing either L1 ORF2 or full length L1 in HeLa ORF2 or HeLa ORF2 ER-- cells. Colony formation was used as a measure of toxicity as previously described [21]. The relative number of G418 resistant (G418^r^) colonies in the two cell lines was measured in triplicate for cotransfection of full length L1 and L1 ORF2. **(B) **Constitutive L1 ORF2 expression in HeLa Cells does not alter sensitivity to ionizing radiation. HeLa ORF2 and HeLa ORF2 ER-- cells were exposed to four different doses of ionizing radiation. After exposure, cells were grown for 3 days and the relative numbers of viable cells were measured. Data are means and SD (error bars) of six independent measurements. Asterisks signify a significant (Student *t*-test, *P *≤ 0.0001) difference from the number of G418^r ^colonies seen in HeLa ORF2 cells. **(C) **Constitutive L1 ORF2 Expression in HeLa cells does not alter cellular proliferation rates. HeLa ORF2 and HeLa ORF2 ER-- cells were seeded, harvested by trypsinization after 1, 2 or 3 days of growth, and viable cells counted. Data are means and SD (error bars) of three independent measurements.

We considered the possibility that the constitutive expression of L1 ORF2 could have caused the HeLa ORF2 cells to grow at an altered rate or otherwise be less fit than HeLa ORF2 ER-- cells, resulting in decreased retrotransposition. To test this possibility, we measured the growth rates of HeLa ORF2 and HeLa ORF2 ER -- cells, and found that their rates of growth were not significantly different from one another (Figure 3A).

The transient expression of L1, and particularly L1 ORF2, is very toxic, resulting in the reduction of cellular proliferation, and induction of apoptosis and cellular senescence [16,17,21]. It is possible that HeLa ORF2 cells had a higher sensitivity to transient expression of L1 ORF2, which could explain the reduced retrotransposition in these cells. To explore this possibility, we measured the toxicity associated with transient L1 expression using a previously described colony formation assay [16]. Expression of either L1 or L1 ORF2 resulted in significantly(*P *≤ 0.05) fewer G418 resistant colonies in HeLa ORF2 ER-- cells than in HeLa ORF2 cells (Figure 3B). This indicates that increased sensitivity to transient L1 expression cannot explain the differences in retrotransposition potential between the two cell lines. Indeed, cells stably expressing L1 ORF2 were more resistant to L1 toxicity.

To determine whether cells constitutively expressing L1 ORF2 were less sensitive to DSBs in general, we measured the toxicity of ionizing radiation (IR) induced DSBs on HeLa ORF2 and HeLa ORF2 ER-- cells. Significantly different sensitivity to IR between these cell lines was not found in either a proliferation or a colony formation assay (Figure 3C, Additional File 3).

Together, these data suggest that the reduced retrotransposition rates observed in the long term L1 expressing cells are not due to an impaired growth rate, and additionally cannot easily be explained by differences in the viability or general health of the cells. The cells seem to have adapted to the stable expression of L1 ORF2 by specifically diminishing their sensitivity to L1 ORF2 as a source of DSBs.

### Repression of retrotransposition is not through RNA interference

**Figure 4:**
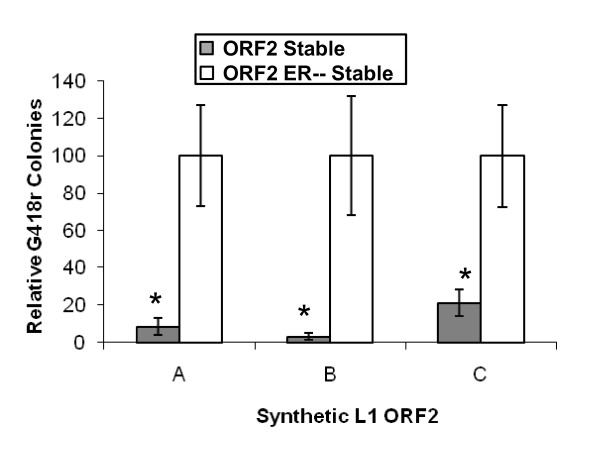
**Repression of Alu Mobilization is not altered by nucleotide sequence variations in L1 open reading frame (ORF)2**. In HeLa ORF2 and HeLa ORF2 ER-- cells, mobilization of a previously described tagged Alu element [45] driven by three synthetic L1 ORF2s [21], each varying in their nucleotide but not amino acid sequence, was measured. Data are means and SD (error bars) of three independent measurements. Asterisks signify a significant (Student *t*-test, *P *≤ 0.005) difference from retrotransposition levels seen in HeLa ORF2 ER-- cells.

Because several of the known activities of L1 ORF2p were repressed by chronic exposure of ORF2p, it was possible that the ORF2 mRNA was being specifically targeted by RNA interference (RNAi) for degradation, a mechanism previously suggested to repress L1 activity [27-30]. To test this possibility, we drove Alu retrotransposition with three synthetic L1 ORF2s containing silent mutations that changed their nucleotide sequence up to 22% but did not modify their amino acid sequence (see Additional file 4). We compared HeLa ORF2 and HeLa ORF2 ER-- cells and found a similar reduction in Alu retrotransposition in both, regardless of which synthetic L1 ORF2 was used. These data suggest that the repressive force that is acting on the L1 ORF2 is not interrupted by large scale changes in the L1 ORF2 mRNA sequences (Figure 4). An even more convincing argument that RNAi is not influencing the retrotransposition rates in these experiments is the fact that the control cells that express mutant L1 ORF2 make the same L1 RNA molecule as the L1 expressing cells, with only a two-base difference. Thus, it is very unlikely that these two almost identical RNAs would not have a similar influence in the RNAi process.

### Constitutive L1 ORF2 expression results in alterations in DSB repair kinetics

**Figure 5:**
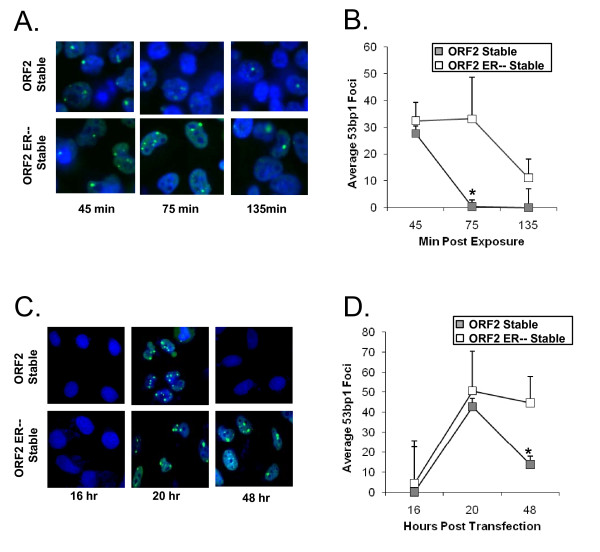
**DSB repair kinetics in cells undergoing constitutive L1 expression**. **(A) **Kinetics of 53BP1 foci formation induced by H_2_O_2_. After treatment with 7.8 mmol/l H_2_O_2_, nuclei of HeLa open reading frame (ORF)2 and HeLa ORF2 ER-- cells were stained with Hoechst and 53BP1 foci are revealed by immunofluorescence. These cells were fixed and stained at three time points (45, 75 and 135 minutes after H_2_O_2 _treatment). **(B) **Quantification of kinetics of 53BP1 foci formation induced by H_2_O_2 _53BP1 foci from (A) were counted. Data are means and SD (error bars) of independent measurements. Asterisks signify a significant (Student *t*-test, *P *≤ 0.05) difference from 53BP1 foci seen in HeLa ORF2 ER-- cells. **(C) **Kinetics of 53BP1 foci formation induced by transient L1 ORF2 expression. After transient transfection with a vector expressing L1 ORF2, nuclei of HeLa ORF2 and HeLa ORF2 ER-- cells were stained with Hoechst and 53BP1 foci are revealed by immunofluorescence. These cells were fixed and stained at three time points (16, 20 and 48 hours after transfection). **(D) **Kinetics of transient 53BP1 foci formation induced by transient L1 ORF2 expression. 53BP1 foci at 16, 20 and 48 hours after transfection were quantified in three separate experiments. Data are means and SD (error bars) of three independent measurements. Asterisks signify a statistically significant (Student *t*-test, *P *≤ 0.055) difference from 53BP1 foci levels seen in HeLa ORF2 ER-- cells.

Because L1 expression generates DNA DSBs that are required for retrotransposition [16], and chronic exposure to DSBs can induce increased DNA repair kinetics in cells [1], we wanted to study the effect of constitutive L1 ORF2p expression on DSB repair kinetics. We measured DSB repair by detecting 53BP1 foci, an early indicator of DSBs, at three time points after exposure to either 7.8 mmol/L H_2_O_2 _or L1 ORF2 expression. 53BP1 foci returned to background levels significantly(*P *≤ 0.05) more rapidly in HeLa ORF2 cells than in HeLa ORF2 ER-- cells after both treatments, indicating that exposure to constitutive L1 ORF2 expression induces a cellular adaptation that more rapidly repairs DSBs induced by exposure to either H_2_O_2 _or transient L1 ORF2p expression (Figure 5).

## Discussion

### Cells adapt to constitutive expression of LINE-1 by repressing Alu and L1 retrotransposition

Human cells adapt to chronic expression of functional L1 elements, specifically the L1 ORF2, in a dose dependent manner. This adaptation includes strong suppression of the retrotransposition potential of both L1 and Alu elements (Figure 2). This is consistent with our observation that retrotransposition was inversely proportional to endogenous L1 expression in cell lines with varying levels of endogenous expression of L1 (Figure 1). It has been found that retrotransposition of human mobile elements is suppressed by a plethora of mechanisms, including DNA methylation, transcription regulation, RNA processing, APOBEC (apolipoprotein B mRNA editing enzyme, catalytic polypeptide-like) proteins and DNA repair pathways [18,23,31-36]. The abundance of methods used to silence these elements is probably necessary because of their high copy number, but also reflects the importance of effective inhibition in almost all cell types and developmental situations. This newly discovered feedback inhibition of L1 ORF2 activity provides a fail safe method of reducing the deleterious effects of L1 ORF2 expression.

**Figure 6:**
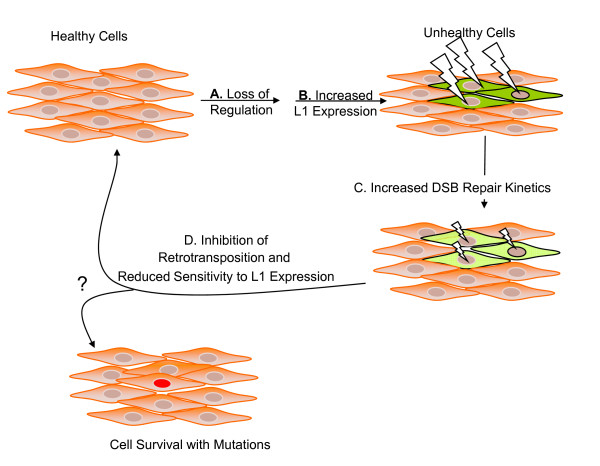
**Model of feedback inhibition of retrotransposition**. In healthy cells, L1 expression is regulated by several mechanisms, but on occasion one or more of these mechanisms can be disrupted, such as loss of methylation induced repression of the L1 promoter associated with early tumorigenesis **(A)**. This loss of regulation of L1 expression is a greatly deleterious condition for cells **(B)**. Cells adapt to constitutive L1 expression by more rapidly repairing double stranded breaks **(C)**. We propose that this increased speed of repair results both in the inhibition of L1 and Alu retrotransposition, and a reduced sensitivity to transient L1 expression **(D)**. Lightning bolts represent toxicity associated with transient L1 expression. Their size represents the relative magnitude of this toxicity. Cells appear green to represent the deleterious nature of L1 open reading frame (ORF)2 expression. Darker shades of green represent cells experiencing greater levels of L1 ORF2-associated toxicity.

Figure 6 outlines how this feedback inhibition would be beneficial to cell growth. In step A, cells are no longer properly repressing L1 expression, perhaps because of the global hypomethylation associated with tumorigenesis [37-41]. In step B, the loss of this repression results in a deleterious increase in L1 expression. In response to this deleterious expression, cells adapt to constitutive L1 expression by increasing the rate of their repair of DSBs, thus reducing the toxicity associated with transient L1 expression (step C). Finally, in step D, the cells repress retrotransposition and minimize the negative effect from insertional mutagenesis and toxicity from L1-induced DSBs.

It is possible that, although it minimizes retrotransposition and direct cellular toxicity, the rapid repair of the DSBs may lead to error prone repair processes. The cellular response to chronic DSBs is to increase NHEJ repair of lesions and hence more rapidly mend the break. Cells exposed to chronic low levels of DSBs induced by L1 also respond by increasing the rate of DSB repair, mostly likely also by increasing NHEJ [1]. Whereas homology independent repair would eliminate the immediate toxic effects of L1 expression, NHEJ is an error prone process that could lead to in increased mutation rate in cells.

### Mechanism of retrotransposition repression by chronic L1 expression

Initially we considered that reduction of retrotransposition might be through an RNAi-like mechanism that responded to the expression of the L1 RNA. However, we dismissed this possibility because the chronic expression of L1 was able to repress Alu retrotransposition, even when an ORF2 with altered nucleotide sequence was used to drive the Alu. More importantly, expression of an inactive L1 ORF2, with only a few point mutations, did not show the same repression (ORF2 ER--). Because the RNA from the mutant ORF2 should only differ by two bases from the wild type RNA, it is very unlikely that it would influence RNAi differentially.

Our data indicate that the most likely mechanism of repression of retrotransposition in our system is a response induced by the DNA DSBs caused by the L1 endonuclease. Constitutive expression of the L1 endonuclease is analogous to the exposure to chronic DSBs, which results in more rapid DSB repair [1]. Although these cells had an altered DNA damage response, they were not more sensitive to ionizing radiation and did not have altered growth rates, just as we detected in response to chronic L1 treatment (Figure 2B). The most notable changes in response to chronic L1 treatment, other than repression of retrotransposition, was that the cells had a significantly (*P *≤ 0.05) faster repair of DSBs and greater protection from toxicity associated with transient ORF2p expression (Figure 5). Thus, we speculate that stimulation of DNA repair allows the cell to clear nascent L1 insertion events before they can complete insertion, much like that demonstrated for the ERCC1/XPF endonuclease [18]. Similarly, it has been suggested that NHEJ proteins limit L1 retrotransposition events by rapidly repairing the L1 generated DSB before the L1 cDNA can be integrated into the break [42].

We found a significant (*P *≤ 0.05) increase in DNA repair associated with a decrease in retrotransposition. The change in DSB repair timing was measured by the formation and clearance of 53BP1 foci. 53BP1 foci format DSBs and are associated with NHEJ mediated repair of these lesions [43]. Because of this association, our data imply an increase in NHEJ repair specifically. Therefore, a likely mechanism for our observed feedback inhibition of retrotransposition is that chronic L1 ORF2 expression induces higher levels of NHEJ that restrict retrotransposition.

We showed that constitutive L1 expression could induce a cellular adaptation that results in more rapid repair of DSBs and repression of retrotransposition. Because it has been suggested that an increase in DSB repair kinetics would decrease retrotransposition, we propose that these two adaptations are related. It is likely that cells adapt to chronic L1 expression by increasing the kinetics of DSB repair, which inhibits retrotransposition. This work highlights the intimate relationship between retrotransposition and DSB repair. In addition, we demonstrate a novel method of cellular adaptation utilized to diminish the toxicity associated with L1 activity.

## Methods

### Northern blots

We combined the contents of four 75 cm^2 ^cell culture flasks of each cell type and extracted total mRNA (TRIzol Reagent, Invitrogen, Carlsbad, CA, USA). We then carried out chloroform extraction and isopropanol precipitation. We selected poly(A) RNA species using a commercial kit (PolyATract mRNA Isolation System III; Promega, Sunnyvale, CA, USA) as instructed by the manufacturer. We resuspended the poly(A) selections and precipitated RNA in 30 μl of RNase-free water and fractionated it in a single lane of agarose formaldehyde gel.

We transferred RNA to a nylon membrane (Hybond-N; Amersham Pharmacia Biotech, Piscatawy, NJ, USA) by capillary transfer overnight at room temperature in a standard 5× sodium chloride/sodium citrate (SSC) solution. We crosslinked the RNA to the membrane with ultraviolet light and prehybridized it in 30% formamide, 1x Denhardt's solution, 1% SDS, 1 mol/l NaCl, 100 μg/ml salmon sperm DNA and 100 μg/ml yeast tRNA at 60°C for at least 6 hours. Hybridization with a strand specific probe was carried out overnight in the same solution at 60°C. We carried out several 10-minute washes at high stringency (0.1x SSC, 0.1% SDS) at 60°C.

We generated (MAXIscript T7 system; Ambion Inc. Austin, TX, USA) the strand specific probe used for the northern blot assay. Primer sequences for generating the template are available on request.

### Retrotransposition assays

Transient L1 [12] or Alu [26] retrotransposition assays were performed as previously described with some minor modifications. Briefly, cells were seeded into T-75 flasks at a density of 5 × 10^5 ^cells per flask. Transient transfections were performed the next day (Lipofectamine and Plus cocktail; Invitrogen), in accordance with the manufacturer's protocol. Cells were grown under selection media containing 400 μg/ml G418 (Geneticin; Fisher Scientific, Pittsburgh, PA, USA) for 14 days. Colonies were fixed, stained and visually scored.

### 53bp1 foci visualization

Cells were plated onto a 96 well imaging plate 16 hours before visualization, and treated with 3.7% formaldehyde followed by 90% cold methanol. After washing, cells were blocked with 1% bovine serum albumin, before being incubated with 2 μg/ml of a 53bp1 antibody (Novus Biologicals, Littleton, CO, USA) for 1 hour. Cells were incubated with 5 μg/ml Alexa 488 conjugated secondary antibodies (Molecular Probes, Eugene, OR, USA) and 5 μg/ml Hoechst stain (Molecular Probes) before being visualized.

### ORF2 western blots

HeLa cells stably integrated with the L1 constructs were grown to confluence in T75 flasks. Cells were treated with trypsin and pelleted at 300 *g*, washed twice with phosphate-buffered saline (PBS), and finally resuspended in 400 uL of PBS. Cells were lysed with an equal volume of 2× Laemmli buffer and boiled for 5 minutes. Extracts were fractionated on a 3 to 8% Tris-acetate gel (Invitrogen) and transferred to nitrocellulose membranes(iBlot System; Invitrogen). Membranes were blocked for 1 hour in 5% milk in PBS-Tween and incubated with α-ORF2 antibody (S19, Santa Cruz Biotechnology) overnight at 4°C. Detection was carried out using horseradish peroxidase conjugated secondary antibodies (Santa Cruz Biotechnology) and a substrate kit (SuperSignal West Femto Maximum Sensitivity Substrate Kit; Thermo Scientific, Waltham, MA, USA). Membranes were exposed to BioMax Light Film (Kodak) and developed with a processor (Kodak X-OMAT 2000A; Rochester, NY, USA). Equal loading was confirmed using β-actin antibodies (Sigma Chemical Co., St Louis, MO, USA) using the same protocol.

### Growth rate

Growth rate was measured as previously described [44] with minor alterations. Briefly, cells were seeded into T-75 flasks at a density of 5 × 10^5 ^cells per flask. After 1, 2 or 3 days of growth, the cells were collected by trypsinization and quantified using a hemocytometer.

### Colony formation assay

Colony formation assays were performed as previously described [21] with some minor modifications. Briefly, cells were seeded into T-75 flasks at a density of 5 × 10^5 ^cells per flask. Transient transfections of a neomycin resistance plasmid were performed the next day (Lipofectamine and Plus cocktail; Invitrogen) in accordance with the manufacturer's protocol. Cells were grown under selection media containing 400 μg/ml Geneticin (Fisher Scientific) for 14 days. Colonies were fixed, stained and visually scored.

### Ionizing radiation colony formation assay

After exposure to ionizing radiation, a colony formation assay was conducted as described both above and in previous works [16].

### Ionizing radiation cellular proliferation assay

After ionizing radiation exposure, the cell growth assay described above was conducted, with the minor alteration that cell growth was measured after 7 days instead of 1 to 3 days.

### Vectors and sequences

All expression vectors used to generate stable cell lines and to drive Alu retrotransposition (including L1 ORF2 A, B and C) were cloned into the pBud CE 4.1 vector by ligation after digestion with *Bam*HI and *Hin*DIII.

The ORF1 sequence of the L1 optimized for expression has previously been described [43]. The L1 ORF2 used in this vector has had synonymous codons replaced with codons of maximum translational efficiency, using a Codon Adaptation Index calculator (http://www.evolvingcode.net) in a manner that preserved the amino acid sequence of the protein.

### Statisical Analysis

In all cases, p-values were determined using the student *t*-test to compare values to the control for each experiment.

## Competing interests

The authors declare that they have no competing interests.

## Authors' contributions

NAW carried out primary studies, including the principle data presented in Figures 2 to 6 and the additional files. NAW also drafted the manuscript. VPB contributed to the manuscript and conducted the studies presented in Figure 1. ZF aided in the characterization of the cell lines. PD participated in the design of the study and its coordination, helping to draft the manuscript. All authors read and approved the final manuscript.

## Supplementary Material

Additional file 1 **Figure 1A**. Cells stably expressing L1 ORF2 have an increased number of endogenous DSBs. **(A) **Nuclei of untreated HeLa ORF2 and HeLa ORF2 ER-- cells were stained with Hoechst, and 53BP1 foci revealed by immunofluorescence. **(B) **53BP1 foci of untreated HeLa ORF2 and HeLa ORF2 ER-- cells were quantified six times. Data are means and SD (error bars) of these six independent measurements. The two cell lines were significantly different (*P *≤ 0.05). **(C) **Western blots with an antibody to the N-terminal portion of ORF2 were carried out on transiently transfected HeLa cells (M), HeLa cells transformed with the L1 endo./RT double-mutant (lane 1; ORF2 ER- in Figure 2B), HeLa cells transformed with ORF2 expression vector (lane 2; ORF2 in Figure 2B) or an optimized L1 vector (lane 3; Opt L1 in Figure 2B) and untransfected HeLa (lane 4). The ORF2 band is marked, as well as a blot of actin on the same membrane. Click here for file

Additional file 2 **Figure 2**. Repression of Alu retrotransposition is not influenced by the source of L1 ORF2. The retrotransposition of a tagged Alu element driven by a vector expressing full length L1 or L1 ORF2 was measured in HeLa ORF2 and HeLa ORF2 ER-- cells. Asterisks signify a statistically significant (Data are means and SD (error bars) of three independent measurements. ≤ 5 × 10^-6^) difference from 53BP1 foci levels seen in HeLa ORF2 ER-- cells. Click here for file

Additional file 3 **Figure 3**. Constitutive expression of L1 ORF2 does not affect sensitivity to 1 Gy of ionizing radiation. The sensitivity of HeLa ORF2 and HeLa ORF2 ER-- cells to 1 Gy of ionizing radiation was measured by transfecting the cells with a neomycin resistance vector after exposure to 1 Gy of ionizing radiation. Data are means and SD (error bars) of five independent measurements. Click here for file

Additional file 4 **Figure 4A, B**. **(A) **Percentage similarity between synthetic L1 ORF2s. Similarity between the synthetic L1 ORF2s is listed in table form. This percentage was calculated as total unchanged nucleotides divided by total nucleotides. **(B) **Sequence alignments of synthetic L1 ORF2s. The row labels refer to the synthetic L1 ORF2 sequence being displayed. (A) signifies L1 ORF2A, (B) signifies L1 ORF2B. C signifies L1 ORF2C. The L1 ORF2 sequences are aligned with L1 ORF2A. Nucleotides matching the L1 ORF2A sequence are shaded. Click here for file
