# Supplementary material for: Feedback inhibition of L1 and alu retrotransposition through altered double strand break repair kinetics
Source: Mob DNA. 2010 Oct 27;1:22. doi: 10.1186/1759-8753-1-22 (PMC3164224; doi:10.1186/1759-8753-1-22)
Supplement: Additional file 1 — Figure 1A. Cells stably expressing L1 ORF2 have an increased number of endogenous DSBs. (A) Nuclei of untreated HeLa ORF2 and HeLa ORF2 ER-- cells were stained with Hoechst, and 53BP1 foci revealed by immunofluorescence. (B) 53BP1 foci of untreated HeLa ORF2 and HeLa ORF2 ER-- cells were quantified six times. Data are means and SD (error bars) of these six independent measurements. The two cell lines were significantly different (P ≤ 0.05). (C) Western blots with an antibody to the N-terminal portion of ORF2 were carried out on transiently transfected HeLa cells (M), HeLa cells transformed with the L1 endo./RT double-mutant (lane 1; ORF2 ER- in Figure 2B), HeLa cells transformed with ORF2 expression vector (lane 2; ORF2 in Figure 2B) or an optimized L1 vector (lane 3; Opt L1 in Figure 2B) and untransfected HeLa (lane 4). The ORF2 band is marked, as well as a blot of actin on the same membrane. [file 1759-8753-1-22-S1.PPT]

## Slide 1
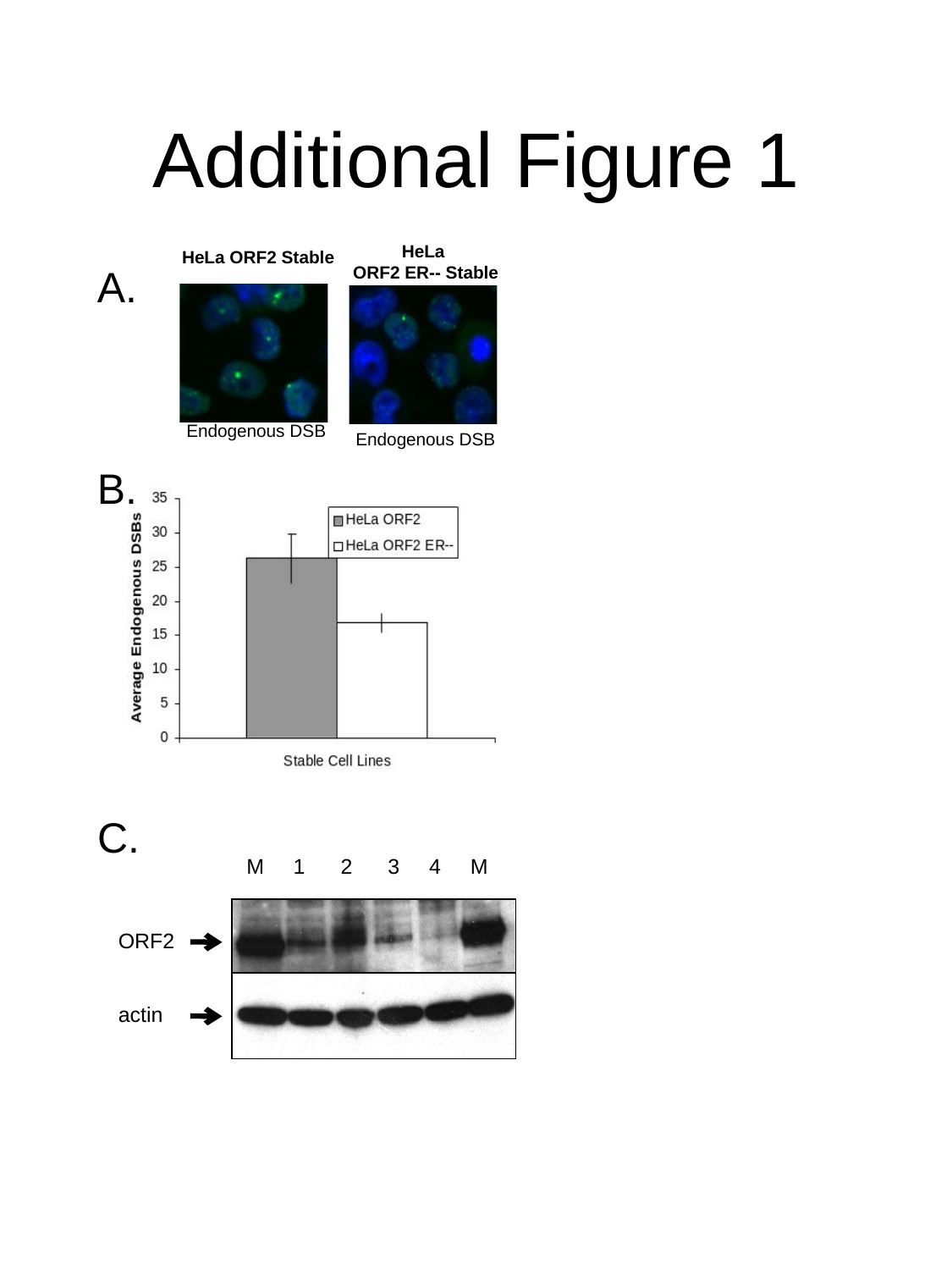

Additional Figure 1
HeLa
ORF2 ER-- Stable
HeLa ORF2 Stable
A.
Endogenous DSB
Endogenous DSB
B.
C.
 M 1 2 3 4 M
ORF2
actin
