# Supplementary figures and images for: Feedback inhibition of L1 and alu retrotransposition through altered double strand break repair kinetics
Source: Mob DNA. 2010 Oct 27;1:22. doi: 10.1186/1759-8753-1-22 (PMC3164224; doi:10.1186/1759-8753-1-22)

## Slide 1
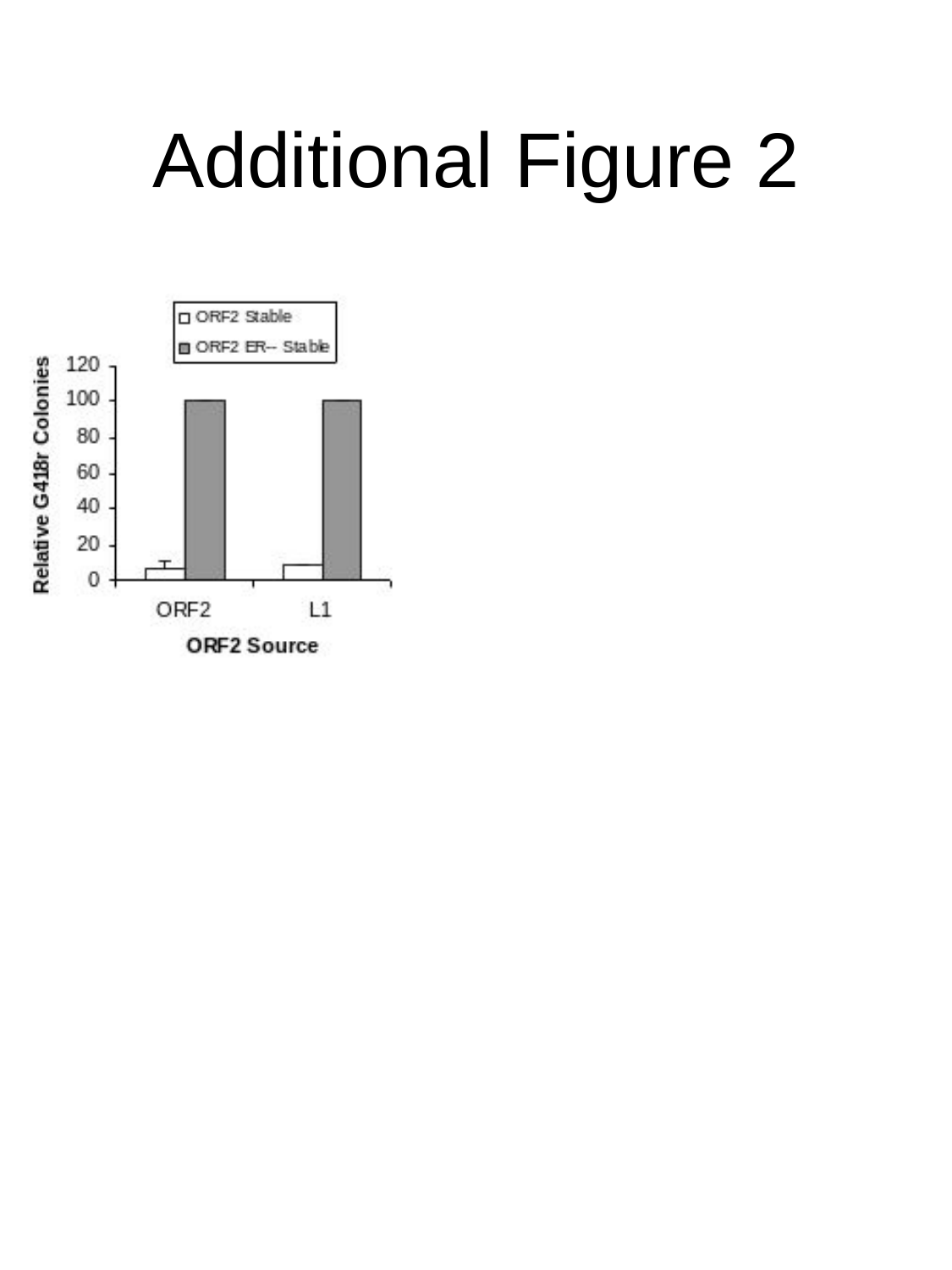

Additional Figure 2

Supplement: Additional file 2 — Figure 2. Repression of Alu retrotransposition is not influenced by the source of L1 ORF2. The retrotransposition of a tagged Alu element driven by a vector expressing full length L1 or L1 ORF2 was measured in HeLa ORF2 and HeLa ORF2 ER-- cells. Asterisks signify a statistically significant (Data are means and SD (error bars) of three independent measurements. ≤ 5 × 10^-6^) difference from 53BP1 foci levels seen in HeLa ORF2 ER-- cells. [file 1759-8753-1-22-S2.PPT]

## Slide 1
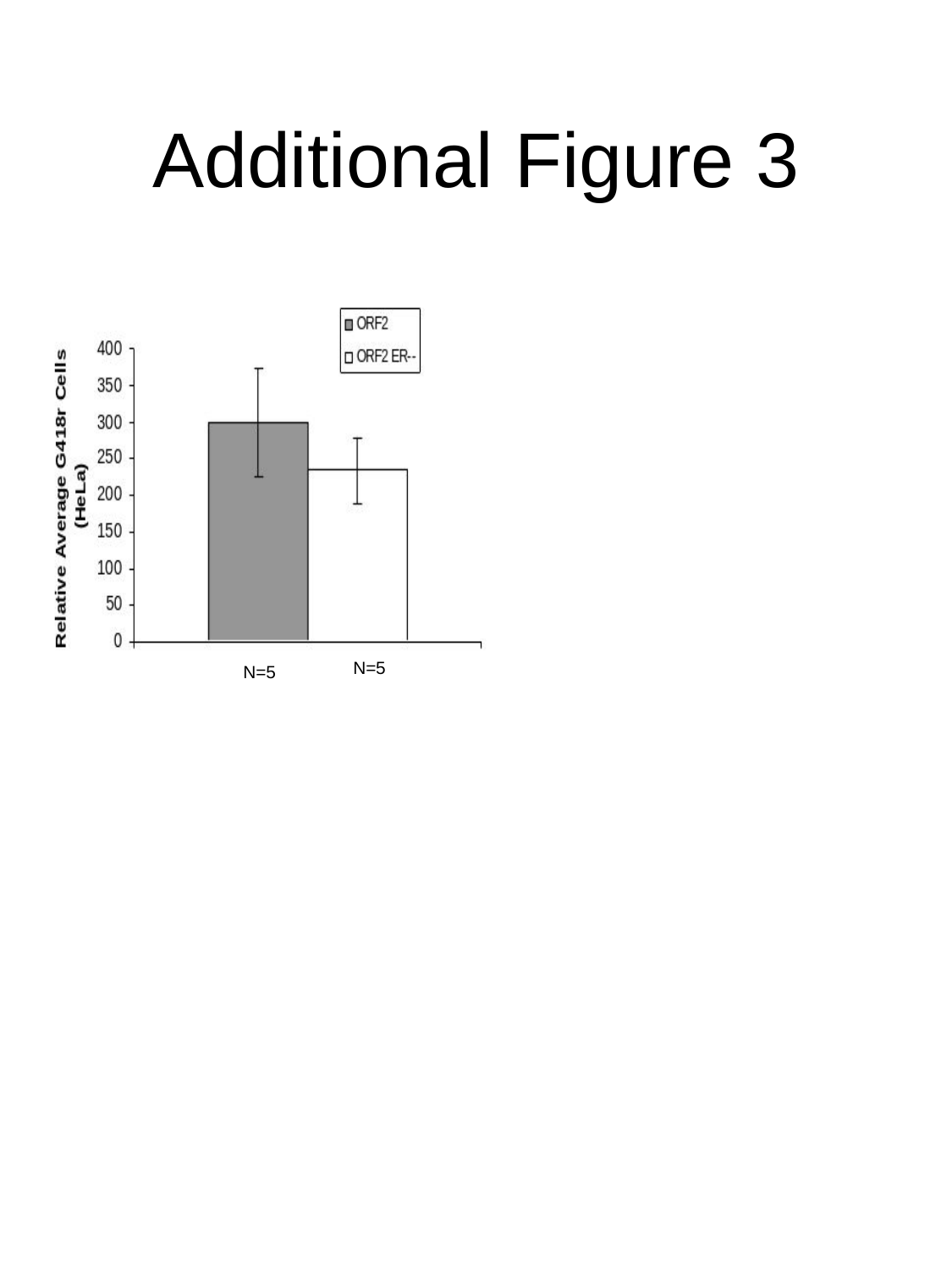

Additional Figure 3
N=5
N=5

Supplement: Additional file 3 — Figure 3. Constitutive expression of L1 ORF2 does not affect sensitivity to 1 Gy of ionizing radiation. The sensitivity of HeLa ORF2 and HeLa ORF2 ER-- cells to 1 Gy of ionizing radiation was measured by transfecting the cells with a neomycin resistance vector after exposure to 1 Gy of ionizing radiation. Data are means and SD (error bars) of five independent measurements. [file 1759-8753-1-22-S3.PPT]
