# Supplementary material for: Feedback inhibition of L1 and alu retrotransposition through altered double strand break repair kinetics
Source: Mob DNA. 2010 Oct 27;1:22. doi: 10.1186/1759-8753-1-22 (PMC3164224; doi:10.1186/1759-8753-1-22)
Supplement: Additional file 4 — Figure 4A, B. (A) Percentage similarity between synthetic L1 ORF2s. Similarity between the synthetic L1 ORF2s is listed in table form. This percentage was calculated as total unchanged nucleotides divided by total nucleotides. (B) Sequence alignments of synthetic L1 ORF2s. The row labels refer to the synthetic L1 ORF2 sequence being displayed. (A) signifies L1 ORF2A, (B) signifies L1 ORF2B. C signifies L1 ORF2C. The L1 ORF2 sequences are aligned with L1 ORF2A. Nucleotides matching the L1 ORF2A sequence are shaded. [file 1759-8753-1-22-S4.PPT]

## Slide 1
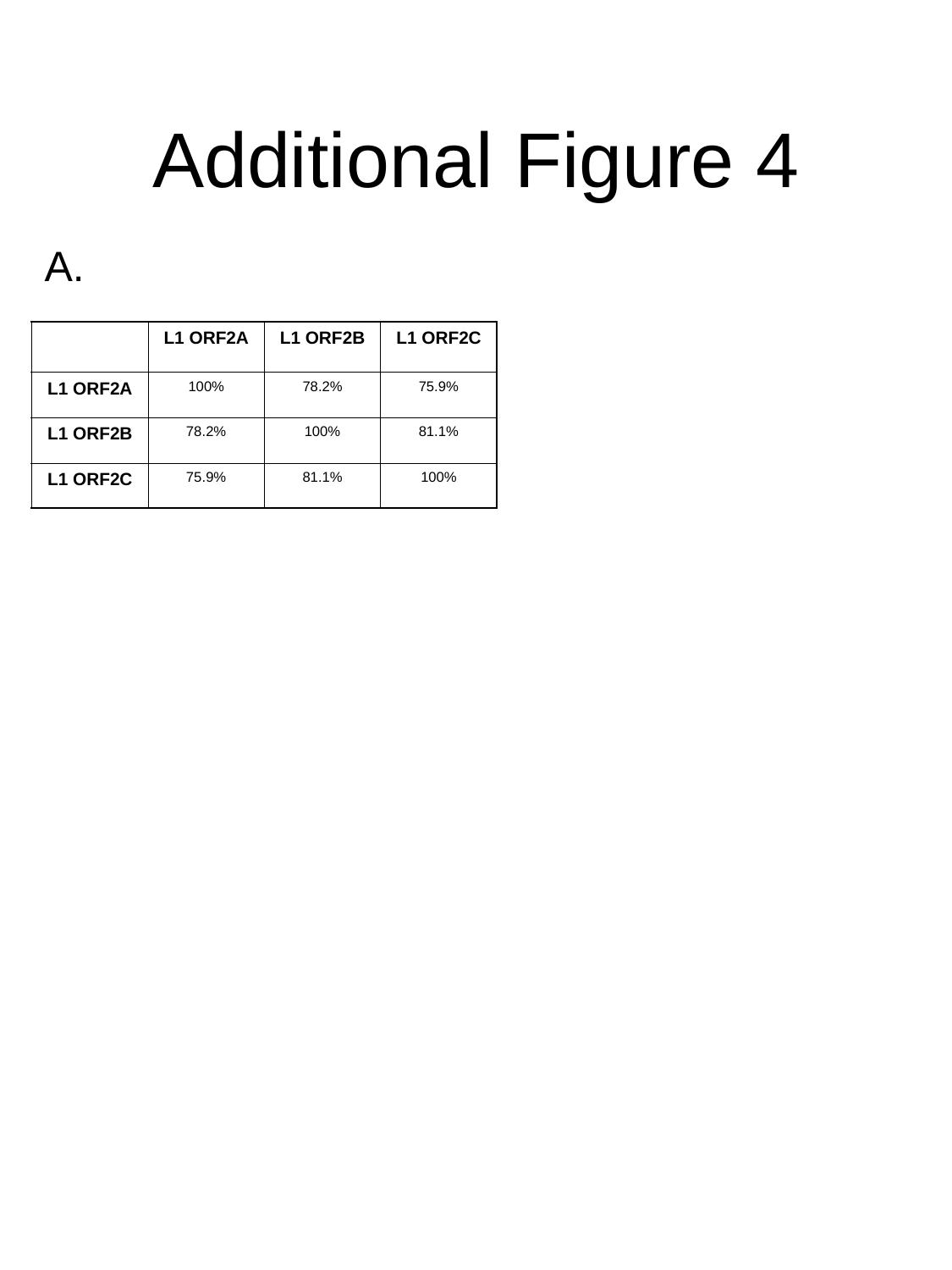

Additional Figure 4
A.
| | L1 ORF2A | L1 ORF2B | L1 ORF2C |
| --- | --- | --- | --- |
| L1 ORF2A | 100% | 78.2% | 75.9% |
| L1 ORF2B | 78.2% | 100% | 81.1% |
| L1 ORF2C | 75.9% | 81.1% | 100% |

## Slide 2
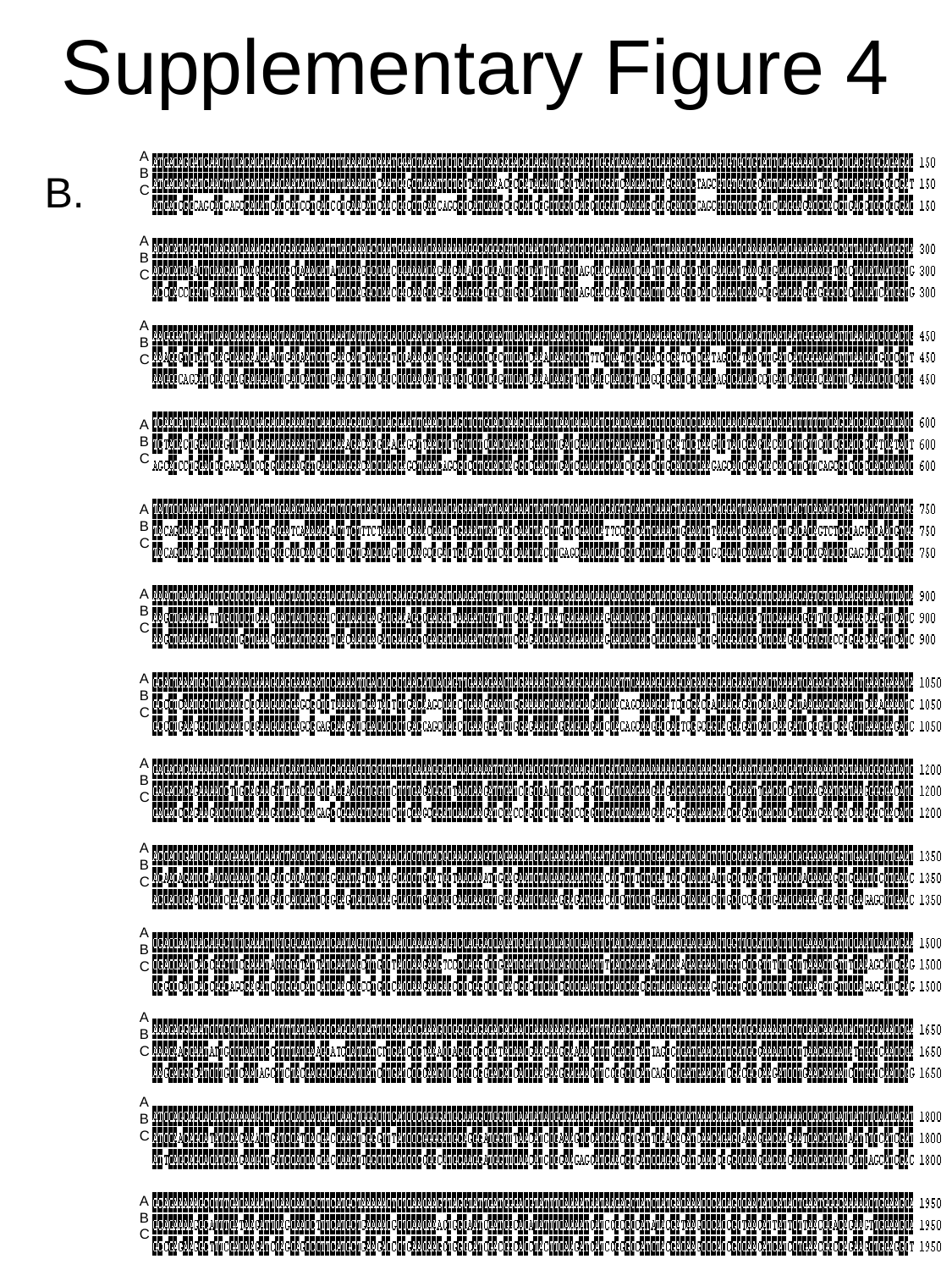

Supplementary Figure 4
A
B
C
A
B
C
A
B
C
A
B
C
A
B
C
A
B
C
A
B
C
A
B
C
A
B
C
A
B
C
A
B
C
A
B
C
A
B
C
B.

## Slide 3
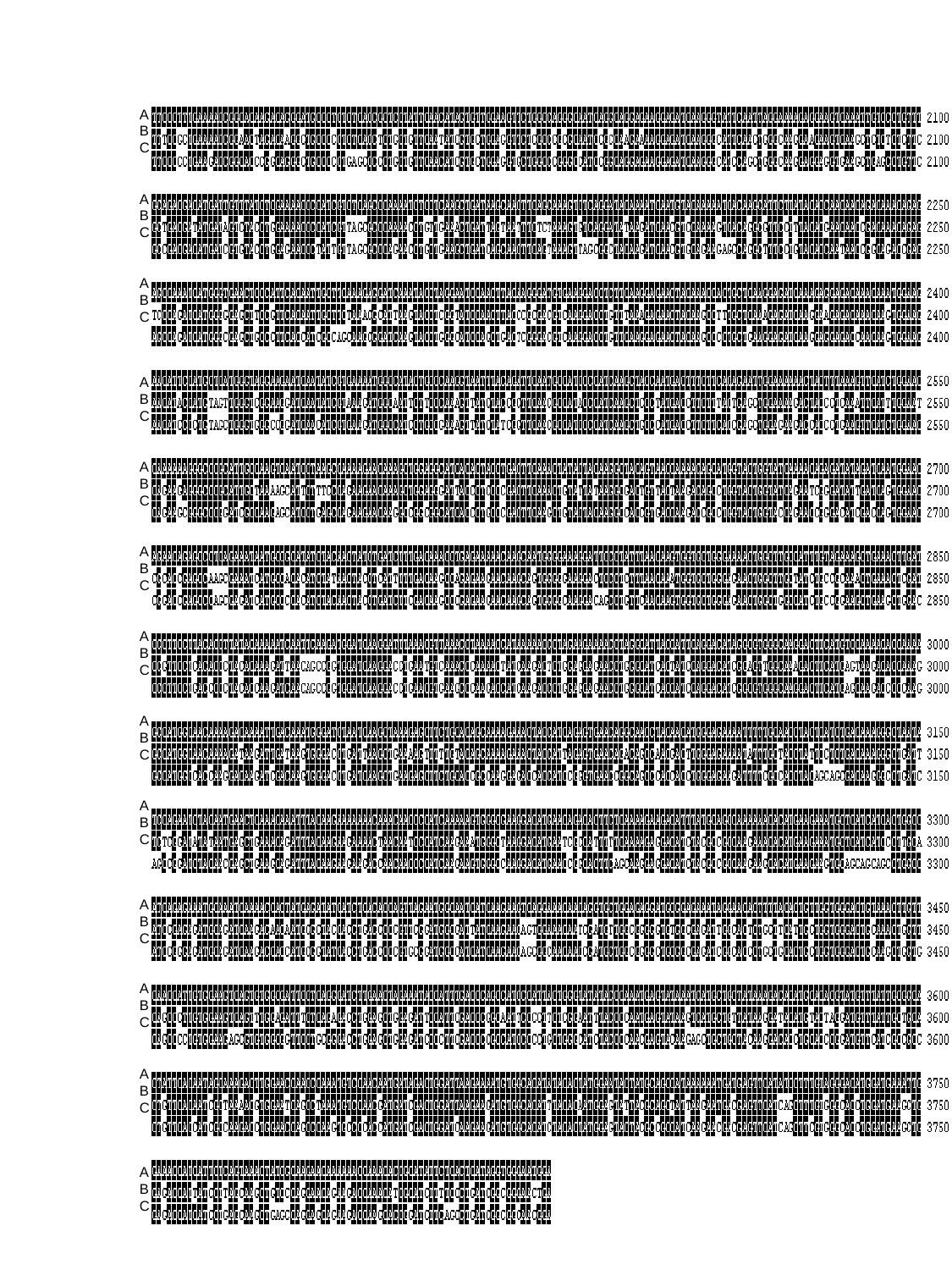

A
B
C
A
B
C
A
B
C
A
B
C
A
B
C
A
B
C
A
B
C
A
B
C
A
B
C
A
B
C
A
B
C
A
B
C
A
B
C
